# Effects of mountain formation and uplift on biological diversity

**DOI:** 10.3389/fgene.2015.00054

**Published:** 2015-02-20

**Authors:** Federico Luebert, Ludo A. H. Muller

**Affiliations:** ^1^Nees-Institut für Biodiversität der Pflanzen Universität BonnGermany; ^2^Departamento de Silvicultura y Conservación de la Naturaleza, Universidad de ChileSantiago, Chile; ^3^Institut für Biologie – Botanik, Freie Universität BerlinBerlin, Germany

**Keywords:** Andes, diversification, orogeny, phylogeny, Tibetan Plateau

The formation and uplift of mountain ranges constitute major geological phenomena that can have long-lasting effects on the evolutionary diversification of living organisms. They provide opportunities for adaptive evolution through an increase of spatial heterogeneity of the landscape, including elevation, and the generation of a wide variety of ecologically diverse biotopes, and affect the migration of organisms and the distribution of species since mountain ranges can act as both biological corridors and ecological barriers. Hence, it should come as no surprise that mountain ranges host a large proportion of the biological diversity on earth (Barthlott et al., 2007; Jenkins et al., 2013). The reviews of Wen et al. (2014) and Luebert and Weigend (2014) included in this Research Topic present accounts of plant diversification processes in two major mountain regions of the world: the Andes and the Qinghai-Tibetan Plateau. Both studies show that plant diversifications have occurred in relatively recent geological times, primarily since the Middle Miocene, and therefore followed the late uplift phases of the high mountain ranges of both the Andes and the Qinghai-Tibetan region during the last 15–20 million years (Garzione et al., 2008; Wang et al., 2008). It is thus likely that the formations of those mountain ranges are at least partially responsible for the observed diversification processes, as proposed in other, more recent studies (e.g., Favre et al., 2015; Sánchez-Baracaldo and Thomas, 2014).

Rapid diversification processes are documented for the Páramo clade of the plant genus *Hypericum* in the northern Andes (Nürk et al., 2013). The high diversity of this group originated recently (2.3–5.6 mya) and its diversification rate is well above the background diversification of *Hypericum*. This pattern of diversification is also seen in other endemic plant groups of the Páramo flora, and the region has the highest average diversification rate among all biodiversity hotspots in the world (Madriñán et al., 2013). Given the similar ages of these lineages (Luebert and Weigend, 2014) and of the high-elevation environments of the Páramo (Mora et al., 2010), these diversifications may have been triggered by the formation of high-mountain habitats in the northern Andes. Increased speciation rates would have occurred along with mountain uplift and habitat diversification, as observed in other Andean groups such as hummingbirds (Chaves et al., 2011) and butterflies (Despland, 2014), confirming the ideas initially proposed by Simpson (1975). Similar diversifications as those observed in the Páramo ecosystem have also occurred in plants of the Qinghai-Tibetan Plateau (Wen et al., 2014), but more studies are necessary to gain insights into any large-scale pattern (Favre et al., 2015).

Mutke et al. (2014) report the distribution patterns of four Andean plant groups to reflect habitat heterogeneity rather than uplift history or barrier effects of mountain ranges, supporting, at least partially, the hypothesis that direct drivers of plant diversification in both the Qinghai-Tibetan Plateau and the Andes include plant-pollinator interactions, local adaptation to diverse environmental conditions and polyploidization (Luebert and Weigend, 2014; Wen et al., 2014). Although not reported in this Research Topic, the occurrence of polyploidization has been shown for the European-centered plant genus *Campanula*. Polyploid species of this genus are concentrated in the *Campanula rotundifolia*-complex, a mountain clade of Pliocene origin (Mansion et al., 2012). The significance of plant-pollinator interactions for the isolation of plant populations and plant diversification in mountain ranges, on the other hand, has been shown for three *Penstemon* species by Kramer et al. (2010).

The different studies reported in this Research Topic clearly illustrate the potential effects of mountain uplift and formation on species diversification, at least in two major mountain regions of the world. A synthesis of biological diversification on mountains is, however, still far from being achieved and the potentially high complexity of the involved history, geography and biological processes encourages further research (Hoorn et al., 2013; Favre et al., 2015; Luebert and Weigend, 2014; Wen et al., 2014). Nevertheless, we hope that the collection of papers in this Research Topic will be of interest to scientists and will stimulate development of new studies and syntheses. We sincerely thank the authors and the reviewers for their efforts and contributions that made this Research Topic possible.

## Conflict of interest statement

The authors declare that the research was conducted in the absence of any commercial or financial relationships that could be construed as a potential conflict of interest.
